# Effect of Ketamine on Human Neurochemistry in Posterior Cingulate Cortex: A Pilot Magnetic Resonance Spectroscopy Study at 3 Tesla

**DOI:** 10.3389/fnins.2021.609485

**Published:** 2021-03-24

**Authors:** Petr Bednarik, Benjamin Spurny, Leo R. Silberbauer, Alena Svatkova, Patricia A. Handschuh, Birgit Reiter, Melisande E. Konadu, Thomas Stimpfl, Marie Spies, Wolfgang Bogner, Rupert Lanzenberger

**Affiliations:** ^1^High Field MR Center, Department of Biomedical Imaging and Image-Guided Therapy, Medical University of Vienna, Vienna, Austria; ^2^Institute for Clinical Molecular MRI in Musculoskeletal System, Karl Landsteiner Society, Vienna, Austria; ^3^Department of Psychiatry and Psychotherapy, Medical University of Vienna, Vienna, Austria; ^4^Department of Medicine III, Clinical Division of Endocrinology and Metabolism, Medical University of Vienna, Vienna, Austria; ^5^Clinical Department of Laboratory Medicine, Medical University of Vienna, Vienna, Austria

**Keywords:** ketamine, glutamate, glutamine, magnetic resonance spectroscopy, depression, ketamine metabolites, posterior cingulate cortex, neurotransmitters

## Abstract

Ketamine is a powerful glutamatergic long-lasting antidepressant, efficient in intractable major depression. Whereas ketamine’s immediate psychomimetic side-effects were linked to glutamate changes, proton MRS (^1^H-MRS) showed an association between the ratio of glutamate and glutamine and delayed antidepressant effect emerging ∼2 h after ketamine administration. While most ^1^H-MRS studies focused on anterior cingulate, recent functional MRI connectivity studies revealed an association between ketamine’s antidepressant effect and disturbed connectivity patterns to the posterior cingulate cortex (PCC), and related PCC dysfunction to rumination and memory impairment involved in depressive pathophysiology. The current study utilized the state-of-the-art single-voxel 3T sLASER ^1^H-MRS methodology optimized for reproducible measurements. Ketamine’s effects on neurochemicals were assessed before and ∼3 h after intravenous ketamine challenge in PCC. Concentrations of 11 neurochemicals, including glutamate (CRLB ∼ 4%) and glutamine (CRLB ∼ 13%), were reliably quantified with the LCModel in 12 healthy young men with between-session coefficients of variation (SD/mean) <8%. Also, ratios of glutamate/glutamine and glutamate/aspartate were assessed as markers of synaptic function and activated glucose metabolism, respectively. Pairwise comparison of metabolite profiles at baseline and 193 ± 4 min after ketamine challenge yielded no differences. Minimal detectable concentration differences estimated with *post hoc* power analysis (power = 80%, alpha = 0.05) were below 0.5 μmol/g, namely 0.39 μmol/g (∼4%) for glutamate, 0.28 μmol/g (∼10%) for Gln, ∼14% for glutamate/glutamine and ∼8% for glutamate/aspartate. Despite the high sensitivity to detect between-session differences in glutamate and glutamine concentrations, our study did not detect delayed glutamatergic responses to subanesthetic ketamine doses in PCC.

## Introduction

Clinical experience confirmed ketamine as a potent tool for the treatment of unipolar depression and bipolar disorders (Kraus et al., 2017b), often effective when other antidepressants fail, i.e., in treatment-resistant major depressive disorder (TRD) (Zarate et al., 2012; Fava et al., 2018; Phillips et al., 2019). Despite that, the exact biological effects of ketamine in the brain remain unknown, and sensitive in vivo metabolic markers of therapeutic drug action still need to be established.

Ketamine’s antidepressant effect is likely mediated through N-methyl-D-aspartate (NMDA) receptors on GABAergic interneurons across cortical regions, where it acts as a glutamate (Glu) antagonist (Abdallah et al., 2015). Thus, ketamine reduces inhibitory control over cortical neurons and indirectly increases cortical activity (Chen et al., 2018) and excitatory glutamatergic neurotransmission in some brain regions such as the prefrontal cortex (Moghaddam et al., 1997). In this regard, increased Glu levels were measured in the cortex activated by physiological stimulus (Bednarik et al., 2015b) and by ketamine challenge (Stone et al., 2012; Milak et al., 2016; Javitt et al., 2018) with single-voxel proton magnetic resonance spectroscopy (^1^H-MRS). Patients suffering from depression had indeed lower cortical glutamate levels due to neuronal loss, decreased neuronal activity, and synaptic dysfunction (Maddock and Buonocore, 2011; Moriguchi et al., 2019) with post-stress dysfunctional glutamate cycling resulting in excitotoxicity and neuronal atrophy (Popoli et al., 2011). Thus, the glutamate model of depression implicates alterations to glutamate-related excitatory synaptic function (Sanacora et al., 2012). Synaptic Glu turnover can be probed by *in vivo* MRS methods quantifying Glu and Gln levels as well as their ratio (Glu/Gln) (Aanerud et al., 2017).

While acute metabolite responses to ketamine administration are more likely associated with dissociative drug effects, antidepressant effects are delayed and start to build-up 2 h after ketamine administration (Berman et al., 2000; Zarate et al., 2006). One of the sparse ^1^H-MRS studies investigating delayed ketamine’s effects revealed a change in the Glu/Gln ratio in the pregenual anterior cingulate cortex (ACC) 24 h after ketamine infusion (Li et al., 2017). The Glu/Gln alterations correlated with delayed ketamine-induced changes in functional connectivity within the default mode network (DMN), including the posterior cingulate cortex (PCC) (Li et al., 2020). Another study showed that connectivity patterns between PCC and regions involved in the DMN (posterior ACC, medial prefrontal cortex), pregenual ACC, and dorsal medial prefrontal cortex were modulated by ketamine (Scheidegger et al., 2012). Although MR imaging connectivity studies show modulatory effects on PCC connectivity, plausible underlying molecular mechanisms in this region have not yet been described. An investigation revealed that patients with major depression had increased resting activity within DMN and PCC compared to healthy individuals (Bartova et al., 2015), which correlated with behavioral measures of rumination and brooding (Berman et al., 2011). These findings were supported by another study, where PCC activity during emotion processing predicted early antidepressant response (Spies et al., 2017). PCC also connects to the hippocampus and might be involved in memory impairment seen in depression (Leech and Sharp, 2014). Hence, PCC dysfunction could also be linked to rumination and memory impairment involved in the pathophysiology of depression. The growing body of ketamine research pointed to the role of PCC in depression and motivated the current study.

Optimized ^1^H-MRS methodology can be used to accurately measure glutamate and glutamine levels. Previous single-voxel ^1^H-MRS studies were mostly focused on ACC, and to our knowledge, none investigated responses to ketamine in the PCC. Therefore, it is pivotal to elucidate whether subanesthetic ketamine administration leads to measurable glutamate and glutamine responses in PCC in the period associated with the presence of antidepressant effects. Such responses could serve as important markers of ketamine’s antidepressant action.

Thus, we utilized the state-of-the-art single-voxel semi-LASER methodology, fine-tuned for highly reproducible measurements at a clinical 3T scanner (Terpstra et al., 2016). Our work aimed to determine the biological *in vivo* effects of the ketamine-challenge on the extended neurochemical profile, including Glu and Gln, in PCC in twelve healthy volunteers.

## Materials and Methods

### Cohort

Twelve healthy male adults (26 ± 5 y.o., mean ± SD) were enrolled in this study. The study population was limited to male participants to avoid oscillations in metabolite levels due to hormonal fluctuations associated with the menstrual cycle (Harada et al., 2011; Liu et al., 2015). Participants were free from internal, neurological, or psychiatric disorders assessed via medical history, physical examination, electrocardiogram, and routine laboratory parameters. Any previous or current psychiatric diagnoses were ruled out by the Structured Clinical Interview for DSM-IV Axis-I Disorders (SCID I) conducted by a trained psychiatrist. Individuals had no history of substance abuse. Current drug use was excluded by urine drug tests performed both at the screening visit and before the MRI sessions. Subjects were excluded at screening if they had any MRI contraindications. All participants provided written informed consent and received financial reimbursement for their participation. This study was approved by the Ethics Committee of the Medical University of Vienna and carried out according to the Declaration of Helsinki.

### Experimental Design

Volunteers underwent two MRI scans. While the first scan (MRI1) was performed without pharmacological challenge and served as a baseline reference, participants received 0.8 mg/kg bodyweight of racemic ketamine (Ketamine hydrochloride, 50 mg/mL ampoules, Hameln Pharma Plus GmbH) intravenously over 50 min starting 193 ± 4 min (∼3 h) prior to the second scan (MRI2). The dose of 0.8 mg/kg is highly efficient to elicit antidepressant effects while kept sub-anesthetic (Perry et al., 2007; Zarate et al., 2012; Fava et al., 2018). Vital parameters were monitored continuously, and a clinician was present at all times. Venous blood samples were drawn at baseline and immediately before and after MRI2. After centrifugation and separation of plasma, samples were frozen at ≤−80°C until analysis.

### MRI/MRS Data Acquisition

**FIGURE 1:**
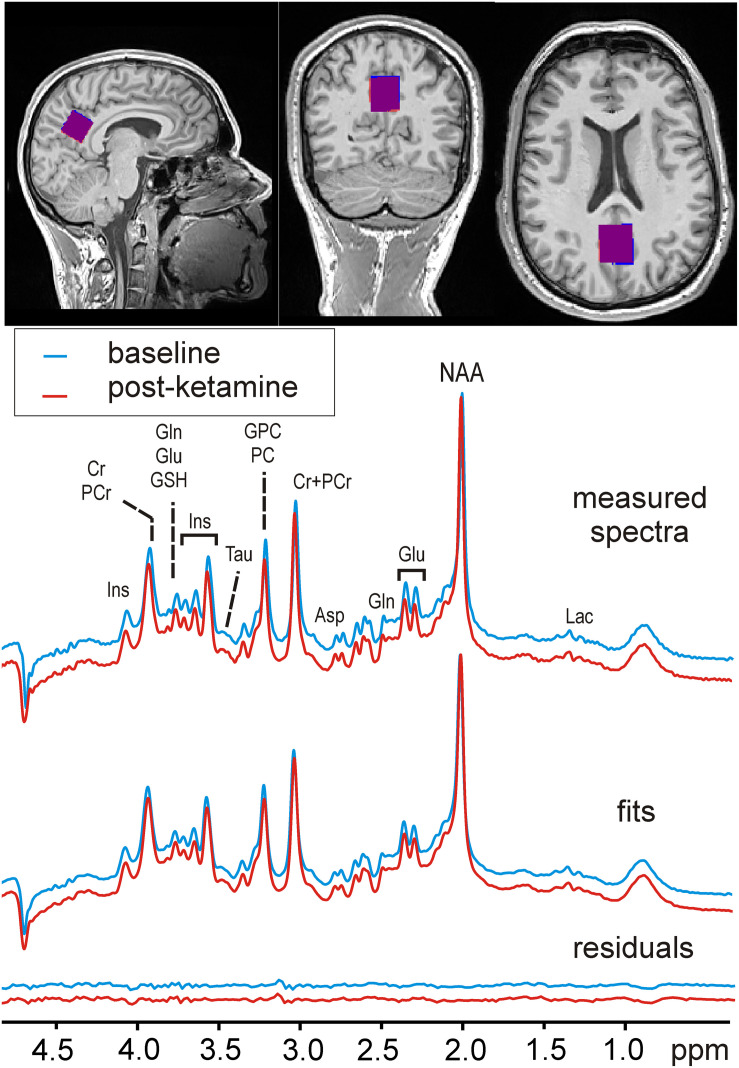
Voxel position and sample MR spectra. An example of spectra acquired before (MRI1) and after ketamine administration (MRI2) is shown along with their fits and residuals of the fits resulting from LCModel quantification. The insets with T1-weighted MPRAGE images depict the typical MRS-voxel position in the posterior cingulate cortex in MRI1 (red) and MRI2 (blue), and their mutual overlap (in purple).

MRI data were collected using a 64-channel head coil on a 3 Tesla MR Scanner (MAGNETOM Prisma, Siemens Medical, Erlangen, Germany). Structural T_1_-weighted images were acquired during each measurement using a standard magnetization-prepared rapid gradient-echo (MPRAGE) sequence (TE = 1800 milliseconds, TR = 2.37 milliseconds, 208 slices, 288 × 288 matrix size, slice thickness 0.85 mm, voxel size 1.15 × 1.15 × 0.85 mm) for accurate placement of the MRS volume of interest (VOI) and within-VOI brain segmentation. The voxel that here we refer to as “Posterior Cingulate Cortex” slightly extended from the posterior cingulate into the parietal lobe (precuneus) but did not extend into the occipital cortex (beyond the occipito-parietal fissure). The 22 × 22 × 22 mm cubic PCC voxel was be placed mid-sagittal on “auto-aligned” anatomical images based on anatomical landmarks (Figure 1). The voxel was rotated in the sagittal plane by ∼30° to be aligned with the posterior border of the splenium. To allow for patient motion and chemical shift displacement, the voxel was backed away anteriorly from the splenium and caudally from occipito-parietal fissure by 2 mm. The precise description of the MRS-VOI position secured its reproducible placement by a single operator (Park et al., 2016).

The position of the VOIs for the post-ketamine MR scan was determined based on the VOI placement of the baseline scan when utilizing Autoalign coordinates (Dou et al., 2015). Standard Siemens B_0_-shimming was used to minimize magnetic field inhomogeneity within the MRS-VOI. MRS data were acquired with a semi-LASER localization pulse sequence (Oz and Tkac, 2011) (128 NEX, TR = 5 s, AT = ∼11 min) with water (Tkác et al., 1999) and outer volume suppression. STEAM based low flip angle water excitation was used to prospectively (each TR) correct for frequency drifts and to keep stable water suppression. Standard full-passage adiabatic pulses were replaced by GOIA-WURST refocusing pulses for optimal localization performance and shorter echo time of 23 ms. Unsuppressed water spectra were acquired as the internal reference for metabolite quantification in absolute units and correction of residual eddy currents.

### MRI/MRS Data Processing

Single-FID MRS data were corrected for small frequency and phase fluctuations, summed and corrected for the residual eddy current effects using an unsuppressed water signal (Klose, 1990). Brain metabolites were quantified by LCModel (Provencher, 1993, 2001; Pfeuffer et al., 1999; Tkáč et al., 2009) using a basis set of nineteen brain metabolites simulated with a spin density matrix approach (Henry et al., 2006), which included: alanine (Ala), ascorbate (Asc), aspartate (Asp), creatine (Cr), γ-aminobutyric acid (GABA), glucose (Glc), glutamate (Glu), glutamine (Gln), glutathione (GSH), glycerophosphocholine (GPC), myo-inositol (Ins), scyllo-inositol (sIns), lactate (Lac), N-acetylaspartate (NAA), N-acetylaspartylglutamate (NAAG), phosphocholine (PC), phosphocreatine (PCr), phosphoethanolamine (PE), and taurine (Tau). Also, a measured spectrum of fast-relaxing macromolecules (MM) was included in the basis set. Only metabolite concentrations quantified with Cramèr-Rao lower bounds (CRLB) below 20% on average were included in further analysis (Terpstra et al., 2016). High-resolution MRI data were used for whole-brain segmentation. The probabilistic maps of the gray matter (GM), white matter (WM), and cerebrospinal fluid (CSF) were calculated with SPM12 (Ashburner and Friston, 2005) from the T_1_-weighted MPRAGE images. An in-house routine written in MATLAB was used to determine the volume fractions of GM, WM, and CSF in each VOI by adopting an iterative method of threshold selection (Ridler and Calvard, 1978). The CSF fraction was used to assess the brain tissue volume for each MRS-VOI. The tissue water content was calculated using GM and WM volume fractions in the VOIs assuming water contents of 84% and 70% in GM and WM, respectively (Randall, 1938; Gröhn et al., 2019). Thus, the CSF fraction and tissue-specific water content were used to adjust metabolite concentrations obtained from each scan. Metabolite concentrations were additionally corrected for T2-relaxation assuming relaxation times of water (T_2_ = 100 ms); this value represents the mean T_2_ from all subjects obtained by fitting the integrals of the unsuppressed water acquired at different TEs with a biexponential fit with the T_2_ of CSF fixed at 740 ms and assuming that the apparent T_2_ of water under Carr-Purcell conditions is 1.5 times that of the measured free precession T_2_ (Deelchand et al., 2018). Finally, the overlap between the pre- and post-ketamine VOI position was evaluated.

### Ketamine and Metabolite Plasma Levels

Determination of ketamine, norketamine (norket), and dyhydronorketamine (dhnk) plasma levels was accomplished using gas chromatography-mass spectrometry (GC-MS/MS) at the Clinical Department of Laboratory Medicine, Medical University of Vienna, Austria. The applied method was validated according to the European Medicines Agency (EMA) guideline on bioanalytical method validation (European Medicines Agency, 2011). Plasma levels were interpolated to the time point in the middle of each MRS measurement for each subject using linear interpolation in MATLAB.

### Statistical Analysis

Data were analyzed in RStudio software version 1.2. All continuous variables were tested for normality using the Kolmogorov-Smirnov test. Signal-to-noise ratios (SNRs) and spectra linewidths provided by the LCModel were first subjected to comparisons with a Wilcoxon signed-rank test to ensure the absence of systematic biases in the datasets induced by distinctions in the data quality pre- vs. post-ketamine. The same statistical test was carried out to compare metabolite concentrations. The significance threshold was adjusted with the false discovery rate method separately for metabolite concentration and ratios to reduce the likelihood of false-positive results to 5%. Results are presented as mean ± SD. To estimate the sensitivity of our method to reveal differences in metabolite concentrations between sessions, the minimal detectable concentration differences were calculated by a post hoc power analysis (power. 0.8, type I error. 0.05), which used the observed standard deviations of the concentration difference (post-ketamine minus baseline).

## Results

The spectra were measured from MRS-VOIs with high between-session overlap 90% ± 5%, with reproducible spectral linewidth 2.5 ± 0.9 Hz and 2.8 ± 1.0 Hz and signal-to-noise ratio 64.5 ± 5.3 and 65.5 ± 5.0 in the first and second scan (*p* > 0.05, respectively. The within voxel fractions of GM (72.4% ± 1.78% and 73.4% ± 1.8%), WM (18.6% ± 3.1% and 17.8% ± 3.2%) and CSF (8.7 ± 3.7% and 8.5 ± 3.1%, session 1 and session 2) were similar in both sessions and the respective between-session coefficients of variations were 1.0% (GM), 4.3% (WM) and 6.7% (CSF) on average.

**FIGURE 2:**
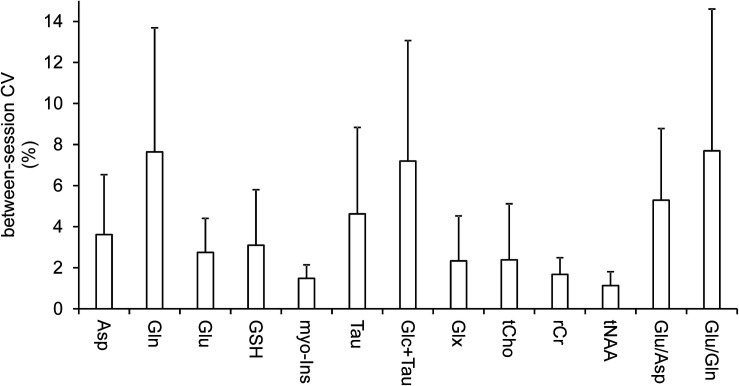
Coefficients of variation. Mean between-session (within-subject) coefficients of variation (CV, SD/mean) illustrate the variance between baseline and post-ketamine scan. Bars are means; error bars represent between-subject standard deviations.

The reproducible quality of measured spectra and their fitting in LCModel along with voxel position is displayed on Figure 1. Six metabolites (Glu, myo-Ins, tCho, tCr, tNAA, and Glx) and macromolecules were quantified with CRLBs below 5%. Another 4 metabolites (Asp, Gln, GSH, Tau, and Glc + Tau) fulfilled the criteria of reliable quantification (CRLB < 20%). Ala, Asc, Glc, sIns, PE, and Lac, had CRLB > 20% consistent with previous literature (Terpstra et al., 2016) and were not analyzed. The mean between-session coefficients of variation (SD/mean) were below 3% for Glu, myo-Ins, tCho, tCr, tNAA, Glx, below 8% for all other reliably quantified metabolites and their ratios (Glu/Gln and Glu/Asp) (Figure 2).

**FIGURE 3:**
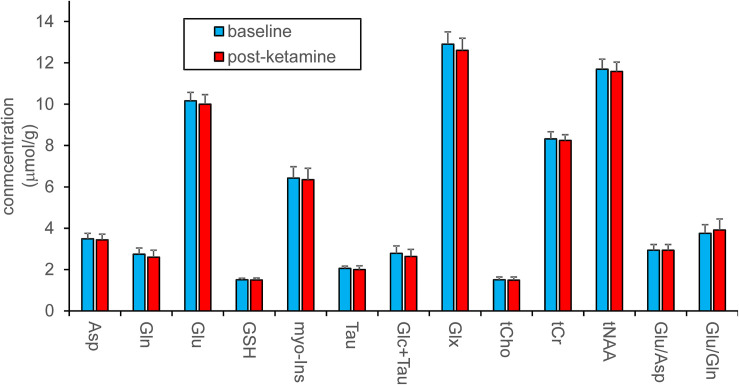
Metabolite quantification in LCModel. Concentrations of metabolites were assessed separately or as sums and are shown in absolute units. Ratios of metabolites are presented in relative units. Data acquired at baseline and 193 ± 4 min. after ketamine administration (*N* = 12) were compared with the standard paired *t*-test, which revealed no differences between pre- and post-ketamine sessions.

**FIGURE 4:**
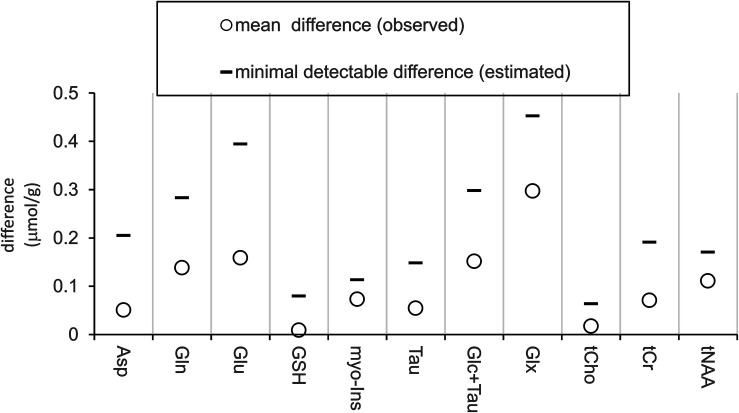
Measured differences and estimated effect size. Plot displays absolute values of measured average differences between sessions (baseline minus post-ketamine, *N* = 12) and minimal detectable differences estimated with power of 0.8 and alpha = 0.05.

Pairwise comparison yielded no statistically significant difference between neurochemical concentrations measured at the baseline and after ketamine administration. Stable neurochemical profiles for both scans are demonstrated in Figure 3. The differences in concentrations (absolute values) between both sessions are substantially smaller than minimal detectable differences estimated by the *post hoc* power analysis (Figure 4). Minimal detectable differences were below 0.5 μmol/g for all metabolites.

Mean plasma concentrations were 77.6 ± 14.7 ng/ml for ketamine, 105.0 ± 21.3 ng/ml for norket, and 21.8 ± 11.15 ng/ml for dhnk. The concentration of ketamine and its metabolites was measured in the middle of the metabolite spectra collection (MRI2), i.e., 193 ± 4 min. after ketamine bolus starts and will serve as a reference for other studies.

## Discussion

MR spectra, obtained in PCC before and ∼3 h after ketamine administration, were referenced to tissue water concentration to reliably quantify 11 neurochemicals in absolute units (μmol/g), and to elucidate delayed ketamine effects in between-session comparison. Ketamine-induced change in Glu and Gln ratio was also assessed (Li et al., 2020). The antidepressant actions of ketamine were previously linked to antagonistic effects on NMDA receptors of GABA interneurons with consequent disinhibition of glutamatergic neurons and glutamate release in some brain regions. Thus, ketamine indirectly activates α-amino-3-hydroxy-5-methyl-4-isoxazolepropionic acid (AMPA) Glu receptor, which triggers second messenger pathways implicated in synaptic function and plasticity (Maeng et al., 2008; Duman et al., 2016; Aleksandrova et al., 2017; Höflich et al., 2017a; Kraus et al., 2017a). Glu, which is involved in several critical metabolic pathways (Mangia et al., 2012), is mostly (80%) created from glutamine by synaptically localized glutamine-synthetase (Tani et al., 2014). Thus, assessing levels of brain glutamate and his precursor glutamine probes the glutamine-glutamate cycle and synaptic functioning (Duarte et al., 2012; Abdallah et al., 2018) and provides insight into ketamine’s antidepressant activity.

Given the dynamic relationship between concentrations of both transmitters via the glutamate-glutamine cycle, their ratio is more susceptible to capture their changes going in the opposite direction, rather than Glu and Gln concentrations assessed separately (Duarte et al., 2012). Besides, we explored another physiologically meaningful ratio, i.e., Glu/Asp, which might reflect deficits in glucose oxidation (Bednařík et al., 2018) reversible by ketamine (Chen et al., 2018). However, in the current study, sub-anesthetic doses of ketamine caused significant concentration changes neither in the separately quantified metabolites nor in the Glu/Gln or Asp/Glu ratios.

PCC is functionally and structurally interconnected to the pregenual ACC, the structure implicated in reward and anhedonia (Höflich et al., 2017b, 2018), the crucial depressive symptom associated with aberrant glutamate metabolism (Walter et al., 2009). Therefore, ACC was most often targeted in the MRS ketamine experiments, which mostly showed an increase in Glu levels after acute ketamine administration and an increase in Gln/Glu 24 h after ketamine infusion. Besides pregenual ACC, PCC connects to other nodes of DMN (dorsal ACC and medial prefrontal cortex, MPFC), and to the dorsolateral prefrontal cortex. An increase in connectivity between these regions seen in depression was reverted by ketamine (Scheidegger et al., 2012). MPFC, along with PCC, are involved in the encoding of negative memories. The functional activation of these structures was linked to the severity of depressive symptoms (Foland-Ross et al., 2013). Another study showed altered connectivity between PCC and lateral orbitofrontal cortex in depression (Cheng et al., 2018). These findings support the theory that the non-reward system in the lateral orbitofrontal cortex has increased effects on the PCC, contributing to the memory system and rumination about sad memories and events in depression (Berman et al., 2011). Overall, PCC is functionally and structurally connected to brain regions involved in affective functions, memory, DMN, and cognition, all related to depression pathophysiology (Leech and Sharp, 2014).

Despite the critical role of PCC in the development and treatment of depressive symptoms, little is known about the underlying molecular mechanisms. To our knowledge, this is the first single-voxel ^1^H-MRS study assessing metabolite response to the ketamine administration in the PCC. To date, only a single multi-voxel 3T ketamine study assessed Glx (Glu + Gln) and GABA concentrations in the voxels located in PCC utilizing edited 3D ^1^H-MRSI methodology (Silberbauer et al., 2020). In agreement with our outcomes, this study revealed no Glx/tCr responses ∼2 h after the ketamine challenge. Although the multivoxel method provided valuable information from other brain regions along with PCC, the work was primarily focused on GABA, and CRLB for Glx in the PCC was ∼9%, i.e., substantially higher than in the current study (CRLB_*Glx*_ = ∼3%). In contrast to our study, the separation of Glu and Gln was not feasible with the multi-voxel approach. In addition, the current manuscript is one of the sparse contributions in ketamine literature that investigates non-acute effects of ketamine on neurochemicals (Li et al., 2017, 2020; Silberbauer et al., 2020).

Moreover, the current study benefited from high within-session (Bednařík et al., 2020) and between-session reproducibility in quantifying neurometabolites (Bednarik et al., 2015a; Terpstra et al., 2016). Notably, the method used in the current clinical trial includes several improvements that were not utilized to obtain data for the methodological test-retest study (Terpstra et al., 2016). Specifically, implemented prospective scan-to-scan frequency correction led to stable water suppression performed with a frequency-selective method, i.e., VAPOR. Additionally, standard HS4 full-passage adiabatic pulses (Deelchand et al., 2014) were replaced with GOIA-WURST pulses resulted in more precise localization of MRS voxel. Lower power requirements of GOIA-WURST allowed shortening of the echo time from 28 to 23 ms (Öz et al., 2020). This governed a slight increase in SNR and a reduction in J-evolution (Landheer et al., 2020). Additional OVS pulses added in the sequence minimized unwanted lipid signals in the spectra. Presumably, these improvements contributed positively to the sensitivity to detect between-session differences in neurotransmitter levels. Indeed, achieved between-session CVs were lower compared to the previous study (Terpstra et al., 2016) and were below 8% and 3% for Gln and Glu, respectively. Nonetheless, this effect can be mainly ascribed to SNR gain owing to larger MRS-VOI (8 mL vs. 10 mL) and more transients (64 vs. 128).

To convey the level of sensitivity at which we ruled out the ketamine effects on neurochemicals, a *post hoc* power analysis was employed to estimate minimal detectable differences between the two sessions (post-ketamine minus baseline). The analysis determined minimal detectable differences of 0.39 μmol/g and 0.28 μmol/g for Glu and Gln, respectively. Translated in relative unites, this indicates, between-session difference of ∼4% in (Glu), ∼10 % in (Gln), and ∼14% in Glu/Gln ratio could be detected in the current study. This sensitivity level allowed to detect functional Glu responses of ∼4% to visual stimulation in the human visual cortex with the same 3T methodology (Bednarik et al., 2019). Authors of another negative MRS ketamine study conducted at 7T estimated their sensitivity to detect Glu responses to ketamine at 8% (Evans et al., 2018). This example emphasizes the critical importance of indicating the power to detect metabolite responses and to utilize optimized methodologies for pharmacological studies.

Despite the high sensitivity of the current methodology, no changes in glutamatergic metabolites in the PCC were observed. In this regard, it is important to note that some studies did not show significant changes in cortical glutamate or glutamine during ketamine challenge in ACC (Taylor et al., 2012; Bojesen et al., 2018; Evans et al., 2018) and OCC (Taylor et al., 2012). This corroborates our recent study utilizing edited 3D MRSI methodology (Silberbauer et al., 2020) that did not prove delayed Glx changes in several brain regions, including ACC and PCC, after ketamine administration. PCC is a structure with complex connectivity and functions; therefore, it is plausible that the ketamine-related metabolite responses could have occured in a subregion of PCC and could not have been revealed due to the partial volume effect given the voxel size of ∼10 mL. It is also possible that delayed changes in Glu and Gln previously detected in ACC (Li et al., 2017) are region-specific and do not occur in PCC. Glutamatergic deficiency in the PCC likely plays a role in acute psychomimetic ketamine effects (Northoff et al., 2005; Ma and Leung, 2018) and can be assessed with sensitive MRS methodologies in the future. For instance, recent advances in 7T MRSI methodology showed excellent Gln and Glu separation and accurate Glu/Gln mapping over the entire brain (Hingerl et al., 2020). Glutamate is coupled to the synthesis of inhibitory neurotransmitter GABA with an important role in depression (Lener et al., 2017). Unfortunately, in the current 3T study, we were not able to quantify GABA with sufficient reliability. However, PCC GABA levels were not disturbed in a previous ketamine study (Silberbauer et al., 2020) utilizing 3D MRSI and spectral editing (Moser et al., 2019; Spurny et al., 2019, 2020). Our study only focused on men population. However, the higher prevalence of anxiety or depression in women than men can be attributed to sex-specific neuronal exposure to different hormonal levels and might cause distinctions in ketamine effects between sexes (Alonso et al., 2004; McHenry et al., 2014). Some animal studies indeed reported distinctions in responses to antidepressants between males and females (Dalla et al., 2010).

Changes in metabolite fluxes are not necessarily reflected by changes in metabolite concentrations. In contrast to ^1^H-MRS, the speed of metabolite turnover and Glu/Gln cycling can be assessed by technically challenging and clinically less accessible ^13^C-MRS (Abdallah et al., 2018). Emerging non-invasive approaches, which benefit from direct and indirect detection of deuterium-labeled tracers with ^2^H and ^1^H-MRS, respectively, promise to provide clinically available quantitative markers of energetic glucose metabolism and synaptic processes that are relevant to ketamine action (Lu et al., 2017; De Feyter et al., 2018; Rich et al., 2020). Also, methods of chemical exchange saturation transfer (CEST) can potentially gain from more sensitive molecular detection of Glc and Glu and can verify our findings in the future (Cai et al., 2012; Roalf et al., 2017; Poblador Rodriguez et al., 2019).

## Conclusion

In conclusion, the current study did not reveal metabolite responses to the ketamine challenge in the period associated with the development of ketamine’s antidepressant action in the posterior cingulate cortex. Specifically, this study ruled out the changes of glutamatergic metabolites, i.e., glutamate and glutamine and their ratio with the respective sensitivity to detect these responses at 4% (Glu), 10% (Gln), and 14% Glu/Gln. While our conclusions might be influenced by the partial volume effect and complex structure of the PCC, quantification outcomes and comprehensive sensitivity analysis will provide valuable information for planning other studies with scan-rescan design.

## Data Availability Statement

The datasets presented in this article are not readily available due to ethical reasons. Please contact petr.bednarik@meduniwien.ac.at for questions.

## Ethics Statement

The studies involving human participants were reviewed and approved by the Ethical Committee of Medical University of Vienna. The patients/participants provided their written informed consent to participate in this study.

## Author Contributions

PB and RL designed the study. PB wrote a draft of the manuscript. BS collected the data. PB, LS, BS, and AS analyzed the data. MS, LS, PH, and MK provided medical support. BR and TS performed the analyses of plasma levels of ketamine and its metabolites. RL and WB were scientific supervisors of the study. All authors were involved in interpretation of the data, critically reviewed and edited the manuscript, and approved its final content.

## Conflict of Interest

RL received travel grants and/or conference speaker honoraria within the last 3 years from Bruker BioSpin MR, Heel, and support from Siemens Healthcare regarding clinical research using PET/MR. RL is a shareholder of the start-up company BM Health GmbH since 2019. The remaining authors declare that the research was conducted in the absence of any commercial or financial relationships that could be construed as a potential conflict of interest.
